# Disparities in lifestyle among community-dwelling older adults with or without mild cognitive impairment: a population-based study in north-western China

**DOI:** 10.3389/fpubh.2025.1533095

**Published:** 2025-05-08

**Authors:** Juxia Zhang, Jiarui Liu, Yuping Feng, Hongyan Meng, Yunhua Wang, Jiancheng Wang

**Affiliations:** ^1^Department of Clinical Education, Gansu Provincial Hospital, Lanzhou, Gansu, China; ^2^School of Nursing, Gansu University of Chinese Medicine, Lanzhou, Gansu, China; ^3^School of Public Health, Lanzhou University, Lanzhou, Gansu, China; ^4^Department of General Practice Medicine, Hospital of Gansu Health Vocational College, Lanzhou, Gansu, China

**Keywords:** cognition, cohort study, lifestyle, mild cognitive impairment, older adults

## Abstract

**Background:**

Evidences indicate that patients with unhealthy lifestyles are at a higher risk of cognitive impairment and dementia. However, uncertainty remains about the association of lifestyles with mild cognitive impairment (MCI) in less-developed areas.

**Methods:**

We used multi-stage stratified sampling method to obtain study population aged ≥65 years, and subsequently a cross-sectional survey was produced including 509 individuals (109 MCI and 400 healthy controls) between March and June 2023. A healthy lifestyle score was defined by scoring six behaviors (non-smoking, non-drinking, exercising, sleeping duration more than 6 h, having a high-quality diet, and controlled BMI). The cognitive function was assessed using the Mini-Mental State Examination (MMSE).

**Results:**

The mean age was 73.8 years, and 57.6% were men of the participates, 21.4% have MCI. Subjects with more healthy lifestyle had significantly lower total score of MMSE, compared to non-MCI subjects. Multivariate logistic regression analysis showed that unhealthy lifestyle behaviors (smoking, drinking, non-compliance diet, harmful sleep, physical inactivity, and harmful BMI) was the significant risk factors for the presence of MCI, independent of factors as sex, age, education level, and family history of AD.

**Conclusion:**

The prevalence of MCI is high, and unhealthy lifestyle is an independent risk factor for MCI in less-developed area. Highlighting the importance of changes in lifestyle behaviors which may influence the cognitive abilities of older adults, specially in settings with approximate conditions.

## 1 Introduction

Worldwide around 50 million people live with dementia, and this number is projected to increase to 152 million by 2050, with around two-thirds of them live in low-income and middle-income countries (1). MCI is the preclinical and transitional stage between healthy ageing and dementia that may be a potential ‘target’ for interventions designed to delay progression to dementia (2). Thus early screening, diagnosis, and intervention for MCI are of great importance, which consequently could theoretically prevent or delay dementia (3). The potential for prevention is high and might be higher in low-income and middle-income areas where more dementia and Alzheimer’ s disease (AD) occur (4). Study highlights rural–urban differences in MCI incidence and access to care, suggesting future research should explore socioeconomic, environmental, and lifestyle determinants of MCI to refine prevention and management strategies across geographic settings (5).

Accumulating epidemiological evidences have shown that healthy lifestyle is a possible risk factor for MCI. A study among stroke survivors found that a healthy lifestyle was associated with a slower rate of cognitive decline (6). Stratified clustering studies reported that a healthy lifestyle correlated with overall cognition, orientation, language ability, delayed recall and executive function (4, 7). Moreover, a study found that lifestyle factors independently contributed to the risk of developing MCI, regardless of genetic risk (8). Furthermore, a systematic review explore the role of lifestyle factors in cognitive health and dementia showed that a healthy diet, and participation in leisure and physical activities may protect against cognitive decline and cognitive impairment, and combined lifestyles may generate multiplicative effects than individual factors (9). However, most existing studies have targeted population in western country or in high-developed cities in China, such as Tianjing (8), Shanghai (4), limited understanding of how combined healthy lifestyle factors would affect cognitive function in individuals of living in underdeveloped areas.

More research is needed to elucidate the causal link between lifestyle and cognition and to better understand the role of healthy lifestyles in the observed associations. Furthermore, data on this topic from underdeveloped regions are important given that increasing trends in dementia coupled with epidemics of MCI can result in upward trends in dementia prevalence and incidence in this setting. Furthermore, the association between lifestyle and MCI may differ in underdeveloped regions due to different culture, and less access to health services, lower education, as well as less resources, inadequate diagnosis and management for dementia, and suboptimal treatment for chronic conditions (10, 11). All these notions underpin the critical needs to understand the dementia related to life-course disadvantage—whether in high or less-developed area. In this context, studying the potential lifestyle factors associated with cognitive function to become a centenarian among those living in underdeveloped areas (12). Therefore, we aimed to investigate whether poor modifiable health behaviors and factors were associated with similar increases in risk of incident MCI among individuals living in Gansu, a less-developed region in Northwest China, where dementia is highly prevalent (35–55% in prevalence) (13) and poorly managed (10, 11). The secondary aim was to investigate possible interactions between health behaviors and MCI.

## 2 Methods

### 2.1 Study participants

The cohort study is an ongoing population-based prospective study focusing on health of older adults in rural areas of northwest China from March 1, 2023, through June 30, 2023. Individuals were included if they met the following criteria: (1) older adults ≥ 65 years old; (2) capable of walking, and with proper vision and hearing to complete the neuropsychological assessments; (3) those who were willing to participate in this study. Multistage stratified cluster random sampling method was used as following steps: first, seven cities were selected from 14 prefecture-level cities based on ethnicity, geographic location, economic level, and total population size. Second, 24 counties were selected from the seven cities based on their population. The average population of Gansu Province in 2022 was used as a criterion. Sample districts (counties) were then chosen, including those with the highest population, the lowest population, and counties with population in the middle. Third, 85 streets (towns) were randomly selected from 337 streets (towns) in the sample counties based on a 25% sampling ratio. Finally, three villages were then randomly selected from each street (town), resulting in a total of 225 villages. A total of 800 qualified individuals were identified. Those willing to participate received general physical examinations and a personal interview conducted by licensed physicians and trained interviewers, respectively. Of the qualified individuals, the exclusion criteria were as follows: recent cardiovascular event (within previous 6 months) (*n* = 60), injury, and trauma (*n* = 117), and missing questionnaire data (*n* = 114). 291 were excluded from this analysis based on the criteria. The remaining 509 participants were included in the analysis, and we identified 109 participants with MCI. Salient characteristics of individuals included and excluded from the current study were largely comparable. The study protocol was approved by the ethics committee of Gansu Provincial Hospital, and all participants provided verbal informed consent before participation. All of our procedures followed the Strengthening the Reporting of Observational Studies in Epidemiology (STROBE) reporting guideline.

### 2.2 Assessment of covariates

The interviewees collected participants’ sociodemographic variables, including age, gender (male or female), educational level (illiteracy, primary school, middle school and above), marital status (married, unmarried), living status and residence (urban or rural) and self-reported or physician-diagnosed hypertension, diabetes mellitus, coronary heart disease, family history of AD.

### 2.3 Assessment of cognitive function

**Figure 1:**
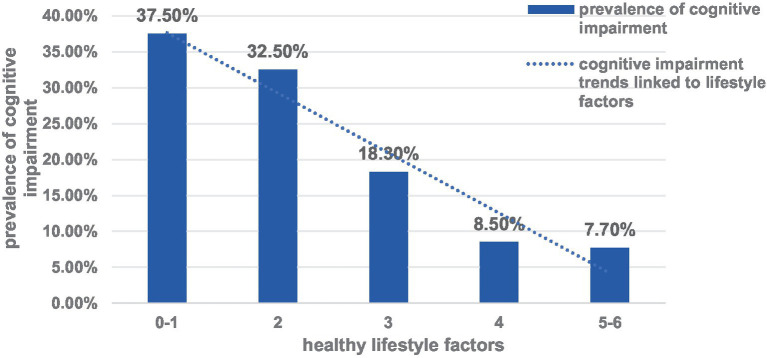
Relationship between healthy lifestyle factors and prevalence of MCI.

The cognitive function of participants was assessed by the Chinese version of the MMSE through a home-based interview, which includes 24 items, covering 7 sub scales including orientation (4 points for time orientation and 1 point for place orientation); naming foods (naming as many kinds of food as possible in 1 min, 7 points); registration of 3 words (3points); attention and calculation (mentally subtracting 3 iteratively from 20, 5 points); copy a figure (1 point); recall (delayed recall of the 3 words mentioned above, 3 points); and language (2 points for naming objectives, 1 point for repeating a sentence, and 3 points for listening and following directions). The MMSE score ranges from 0 to 30. Higher scores represent a better cognitive function. The validity and reliability of this Chinese MMSE has been verified in several previous studies, with an inter rater correlation coefficient of 0.998 (14). Considering the significant correlation between education level and MMSE scores and according to the results of previous literature, the criteria for the mild cognitive impairment group are as follows:

The older adults with illiterate education level scored 17 points in MMSE;
The older adults with primary school education have an MMSE score of 20 points;
The older adults with secondary education or above had an MMSE score of 24.

### 2.4 Construction of healthy lifestyle score

Based on the evidence (15, 16), guidelines (17), and expert knowledge, we constructed a healthy lifestyle score with six healthy lifestyle components, including smoking, alcohol drinking, physical activity, dietary diversity, sleep duration, and BMI (12). Data were collected through questionnaire interviews.

In brief, participants were asked about (1) their smoking status (yes/no), with non-smokers get 1 point. (2) alcohol drinking (yes/no), with drinking less than once a week, drinking weekly but not daily, or drinking daily, men’s intake of pure alcohol was < 30 g or women’s < 15 g get 1 point. (3) In terms of physical activity, participants were asked about whether there is a regular exercise: exercise at least 3 times a week for at least 30 min each time (yes/no). (4) Dietary information was collected according to adherence to the Mediterranean diet pattern, using a 14-point screening scale, the total score of the dietary pattern score was the sum of adherence to the Mediterranean diet pattern, and the diet score was measured by the food frequency questionnaire; Scores range from 0 to 14. Score ≥10: compliance. Participants got 1 point for compliance, otherwise, scored 0 (18). (5) Previous studies have shown a significant association between sleep duration and cognitive function and dementia risk. Maintaining 6–7 h of sleep duration is important to optimize cognitive performance. Therefore, according to the length of sleep, the patients were divided into three groups: “< 6 h,” “6-7 h” and “>7 h.” < 6H and >7 h were unfavourable without score, and 6 h-7 h scored 1 point (19). (6) Dietary guidelines for Chinese residents (2022) state that the appropriate body weight and BMI for people over 65 years of age should be slightly higher than that of ordinary adults, ranging from 20 to 26.9 kg/m^2^. Those with a BMI in the normal range get 1 point, otherwise no score.

The healthy lifestyle score was created by a composite of the six healthy lifestyles based on previous studies (19). Overall healthy lifestyle score was the sum of the individual scores of all six healthy lifestyles, ranging from 0 to 6 points, with a higher score indicating a healthier lifestyle. Participants were categorized into five groups using the cutoff values of healthy lifestyle score following the suggestions from similar studies in the literature: 0–1, 2, 3, 4, 5–6 (20).

Participants were categorized into two groups according to MMSE score (with MCI or without MCI). Baseline characteristics were described across these two groups. Lifestyle score was examined as a continuous variable with a one-point increase (e.g., one additional healthy factor) in the lifestyle score (0–6) and as a categorical variable in which we grouped study participants into five groups, 0–1, 2, 3,4, and 5–6 healthy (i.e., low-risk) factors with the reference category those with 0–1 healthy lifestyle factor.

### 2.5 Statistical analysis

Models were adjusted for age, sex, race, education, and comorbidities. Categorical variables were summarized as frequencies and percentages, and continuous variables as means±standard deviations. ANOVA for continuous variables and chi-squared tests for categorical variables were applied to test the significance levels of the differences and subsequently, binary Logistic regression were employed to examine the associations of healthy lifestyle with risk of CI with participants following unhealthy lifestyle as the reference group.

Results were reported as Odds ratios (OR) and corresponding 95% confidence interval (CI). We adjusted the demographic and socioeconomic characteristics, i.e., age, sex, education, annul household income, and a history of hypertension and diabetes. To examine the interaction between MCI and healthy lifestyles, subgroup analyses stratified by lifestyle factors were conducted.

All analyses were adjusted for confounders (i.e., age, sex, education, annual household income, BMI and history of hypertension and diabetes) and performed in SPSS version 27.0 and R 4.4.1. A two-sided *p* < 0.05 was considered statistically significant.

## 3 Results

### 3.1 General information

Table 1 presents the demographic and lifestyle characteristics of participants. A total of 509 participants, including 109 identified MCI and their matched 400 healthy controls, with the incidence of MCI 21.4%. Of this sample, the mean age was 73.8 (4.3) years, with 290 (57.6%) were men. About 9.1% of participants in the MCI group were current smokers and only 6.1% engaged in routine physical activities. No significant differences were detected between MCI and controls group regarding their sociodemographic characteristics.

**Table 1:** Demographic and lifestyle characteristics of study population (*n* = 509).

Characteristics	Categories	Total (*N* = 509)	Without MCI (*n* = 109)	With MCI (*n* = 400)	χ2/F	*p*
Gender	Male	293	233 (45.8)	60 (11.8)	0.360	0.549
	Female	216	167 (32.8)	49 (9.6)		
Residence	Urban	342	272 (53.4)	70 (13.8)	0.555	0.456
	Rural	167	128 (25.1)	39 (7.7)		
Age	–	509	400 (72.25 ± 7.16)	109 (74.67 ± 6.77)	10.007	**0.002**
Education	Illiteracy	68	44 (8.6)	24 (4.7)	9.116	**0.010**
	Primary	92	73 (14.3)	19 (3.7)		
	Middle school and above	349	283 (55.6)	66 (13.0)		
Family history of AD	Yes	14	8 (1.6)	6 (1.2)	3.933	**0.047**
	No	495	392 (77.0)	103 (20.2)		
Hypertension	Yes	240	179 (35.2)	61 (12.0)	4.322	**0.038**
	No	269	221 (43.4)	48 (9.4)		
Diabetes	Yes	154	111 (21.8)	43 (8.4)	5.556	**0.018**
	No	355	289 (56.8)	66 (13.0)		
Coronary heart disease	Yes	99	82 (16.1)	17 (3.3)	1.315	0.252
	No	410	318 (62.5)	92 (18.1)		
Healthy lifestyle factors						
Smoking	Yes	174	128 (25.2)	46 (9.1)	4.237	**0.040**
	No	334	272 (53.5)	62 (12.2)		
Alcohol consumption	Yes	137	98 (19.3)	39 (7.7)	5.540	**0.019**
	No	372	302 (59.3)	70 (13.8)		
Physical activity	Yes	225	194 (38.1)	31 (6.1)	13.975	**<0.001**
	No	284	206 (40.5)	78 (15.3)		
Sleep duration	Beneficial	73	65 (12.8)	8 (1.6)	5.536	**0.019**
	Harmful	436	335 (65.8)	101 (19.8)		
Dietary pattern	Compliance	48	47 (9.2)	1 (0.2)	11.769	**<0.001**
	Non-compliant	461	353 (69.4)	108 (21.2)		
BMI	Beneficial	381	311 (61.1)	70 (13.8)	8.330	**0.004**
	Harmful	128	89 (17.5)	39 (7.7)		

MIND, Mediterranean-DASH Diet Intervention for Neurodegenerative Delay diet score; AD, Alzheimer’s disease. The bold values indicate *p* < 0.05, with statistical significance of the differences.

### 3.2 Association of healthy lifestyles factors with prevalence of MCI

Figure 1 showed that participants with more healthy lifestyle factors had a lower prevalence of MCI. Among the lifestyle factors, only 117 participants (23%) met at least 4 of the 6 healthy lifestyle criteria. Using healthy lifestyle factor as the independent variable and cognitive impairment as the dependent variable. Adjusted factors: age, gender, educational level, family history of dementia, hypertension and diabetes. Binary Logistic regression results showed that having a more healthy lifestyle factor was associated with a lower prevalence of MCI, with or without adjustment for sociodemographic characteristics. Compared with participants with 2 healthy lifestyle factors, the adjusted OR and 95% CI for participants with 4, 5–6 factors were 12.27 (95% CI, 4.88–30.82), and 14.22 (95% CI, 2.86–70.59), respectively (Table 2).

**Table 2:** Adjusted odds ratios for the association of lifestyle with MCI (*n* = 509).

Healthy lifestyle	Without MCI (*n*, %)	With MCI (*n*, %)	Unadjusted		Adjusted	
			OR (95%CI)	*P*	OR (95%CI)	*p*
0–1	45 (62.5)	27 (37.5)	Ref.	–	Ref.	–
2	77 (67.5)	37 (32.5)	1.24 (0.67–2.31)	0.481	1.67 (0.86–3.24)	0.128
3	147 (81.7)	33 (18.3)	2.67 (1.45–4.91)	**0.002**	5.23 (2.50–10.92)	**<0.001**
4	107 (91.5)	10 (8.5)	6.42 (2.87–14.35)	**<0.001**	12.27 (4.88–30.82)	**<0.001**
5–6	24 (92.3)	2 (7.7)	7.20 (1.57–32.89)	**0.011**	14.22 (2.86–70.59)	**0.001**

^*^Models adjusted for Age, Sex, Education level, Family history of Alzheimer’s disease, Hypertension, Diabetes. CI, confidence interval. The bold values indicate *p* < 0.05, with statistical significance of the differences.

### 3.3 Association of lifestyle behaviors with MCI

**Figure 2:**
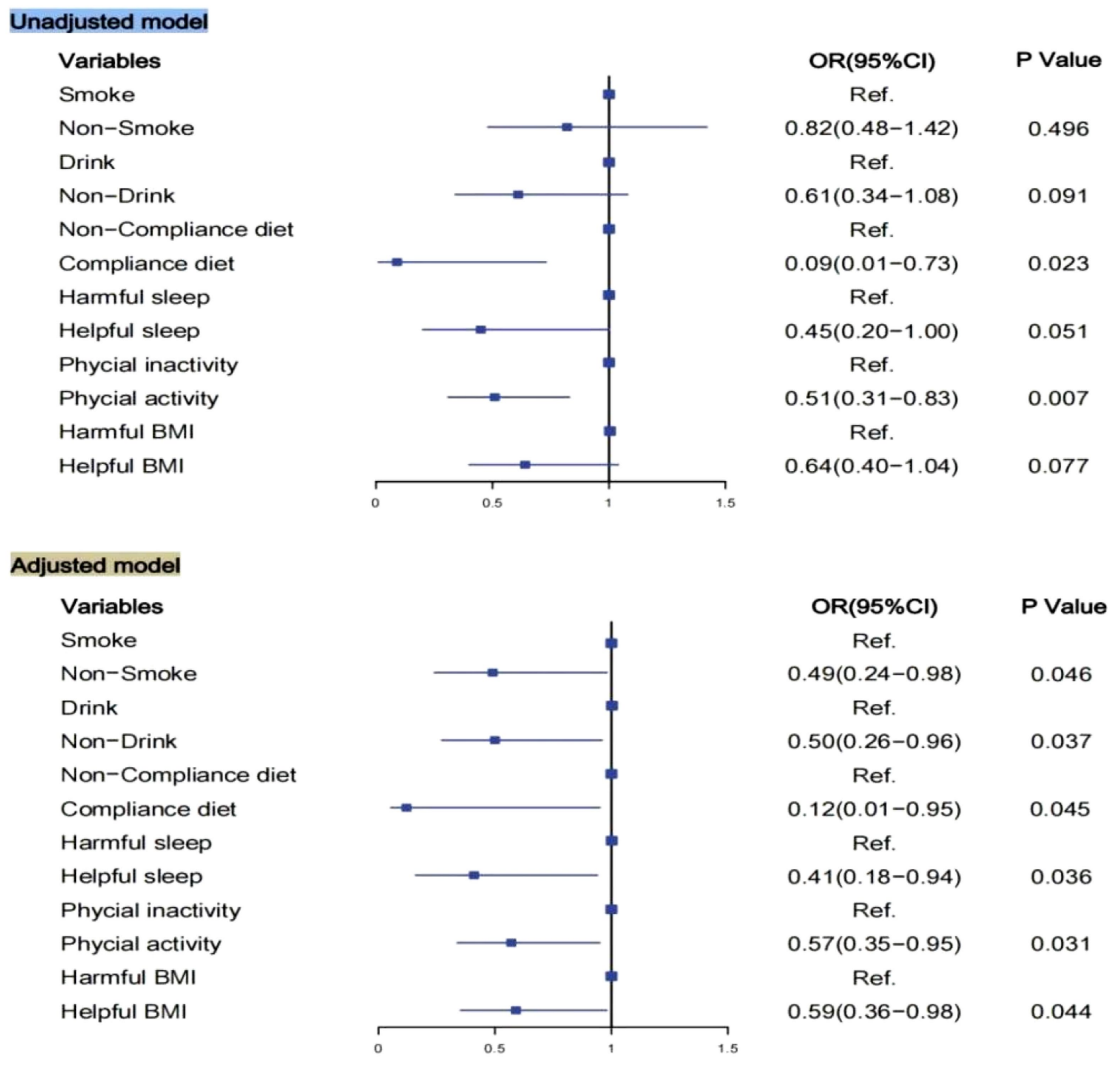
Logistic regression model for association of lifestyle and MCI. Models adjusted for Age, Sex, Education level, Family history of Alzheimer’s disease, Hypertension, Diabetes. BMI, body mass index; OR, Odds ratios; CI, confidence interval.

Figure 2 shows that an unhealthy lifestyle behaviors (smoking, drinking, non-compliance diet, harmful sleep, physical inactivity, and harmful BMI) was significantly associated with prevalence of MCI in the univariable model, adjusting for sociodemographic characteristics (Age, Sex, Education level, Family history of Alzheimer’s disease, Hypertension, Diabetes). Of the lifestyle factors, healthy diet and helpful sleep are of utmost importance, considering their greater ORs.

## 4 Discussion

The findings of this population-based cohort study show that unhealthy lifestyle has a positive impact on cognitive function. Study participants with MCI had a significant more unhealthy lifestyle than those without, creating a “positive circle” that further promotes cognitive function over time. In other words, it was found that changes in lifestyle behaviors predicted changes in cognition and an individual’s cognitive ability predicted their lifestyle. The findings of the adjusted covariates (demographic characteristics) suggest that individuals may experience a subsequent increase in cognitive function when engaging in more health lifestyle than usual.

Notably, we found that 21.4% of elder adults in our sample had MCI, representing 71.7 million adults aged 65 years or older in Gansu, China, higher than most previous estimates. A relatively large-sample studies in 2018 reported the overall MCI prevalence was estimated to be 15·5% (15·2–15·9) in Chinese people aged 60 years or older (21). However, results in English Longitudinal Study on Ageing, reported that 23.4% of participants were affected by MCI (22). A study in West Michigan (423,592 patients), reported a higher MCI incidence in urban settings compared to rural settings (38.3% vs. 32.2%) (5). The differences in prevalence might be explained by different cognitive management, and medical resources preceding the onset of MCI. Moreover, heterogeneity in research methods, including the use of different diagnostic criteria, can affect results. Unfortunately, most people with MCI were undiagnosed, and inadequately treated. A study in the U.S. found that although detection rates for MCI cases increased over time, 92% expected MCI cases remained undiagnosed, particularly in socioeconomically disadvantaged groups (23). Study in West Michigan also suggests potential underdiagnosis in rural areas likely due to reduced access to specialists (5). Most patients with MCI even did not know what MCI was, and did not realize that it could progress into dementia in China (21). Additionally, few reports have focused on the management of MCI, making this a poorly understood, yet crucially important, topic. MCI has a greater risk of progression to dementia, which depends on factors such as sample size, geographic region, the nature of individuals, cultural background, length of follow-up (21). A prevention strategy should be developed to target the identified risk factors in the MCI population to thwart or slow down disease progression, especially in less developed region.

Among the lifestyle factors, only 23% of participates met at least 4 of the 6 healthy lifestyle criteria. This percentage is quite low and consistent with previous study from China (24). Previous meta-analyses show that combinations of at least three unhealthy lifestyle are associated with more than twice the risk of all-cause, cardiovascular disease, and cancer mortality (25). As unhealthy lifestyle tend to cluster together - individuals with one unhealthy lifestyle often have more than one (26). Our results can deepen understanding of the distribution of these lifestyle-related adverse health outcomes among populations living in less developed region. As with cognitive outcomes follow clear and long-recognised healthy lifestyle gradients where individuals of less developed region (e.g., those with lower educational attainment, lower income, or who live in areas of higher deprivation) tend to have higher rates of cognitive impairment. Whats more, healthy diet is of utmost importance, considering the greater ORs in this study. This phenomenon is a clear signal that more attention should be paid to diet-related knowledge of older adults living in less developed region. Evidence showed that individuals’ knowledge and attitudes are implicated in achieving desirable dietary behavioral changes, which, in turn, lead to better health outcomes (27). Specifically, individuals who identify barriers associated with their attitude towards healthy eating are more likely to have a poorer quality diet (28, 29). The high cost of access to diverse food, such as transportation and electricity costs, could be a possible explanation for regional differences (30). Previous systematic review has shown that individuals with more comprehensive dietary knowledge tend to engage in healthier eating patterns (31, 32). Thus, there is an opportunity to provide dietary interventions and dietary guidelines that are more desirable and potentially sustainable for elder adults in less developed region, which could facilitate the acquisition of essential health-related knowledge and strengthen motivation to engage in healthy dietary behaviors. In addition, details about the mean lifestyle across household structures in urban and rural areas should be explored, so as to find out the effectiveness of lifestyle on cognitive function within families. Whats more, we found helpful sleep, no smoking, and limited alcohol consumption should be promoted vigorously to help to prevent MCI.

## 5 Limitations

This study had several limitations. First, lifestyle factors were collected by questionnaire. Second, other factors may also contribute to the development of MCI besides lifestyle; study of those factors can further improve the accuracy of risk. Third, the study sample comprised participants of the cohort in Gansu, china; therefore, further studies are warranted to determine the extent of extrapolation of these results to populations in other less developed region. Furthermore, conclusions based on a 1-time survey may be underestimating the extent to which elder adults experience MCI throughout lifespan, an extended follow-up period are needed to further validate the current findings.

## 6 Conclusion

In this cohort study, we conducted an in-depth analysis focusing on the older adults population in less-developed area, revealing that unhealthy lifestyles are an independent risk factor for MCI. This finding not only deepens our understanding of the complex mechanisms underlying cognitive decline in the older adults but also emphasizes the significance of modifications in lifestyle behaviors for preserving cognitive abilities, particularly in areas with comparable resource and environmental conditions.

From a professional perspective, the decline in cognitive function is a multifaceted outcome influenced by numerous factors, including but not limited to genetic predispositions and lifestyle. This study focuses on the controllable factor of lifestyle, discovering that unhealthy lifestyle can significantly increase the risk of MCI among the older adults. This discovery resonates with existing extensive epidemiological and biological evidence, further confirming the pivotal role of lifestyle in maintaining cognitive health. From a practical standpoint, this study holds significant implications for public health policy formulation and community health management in less-developed regions. In these areas, due to relatively limited economic conditions and medical resources, prevention strategies often rely more heavily on individual self-management. Therefore, based on the results of this study, it is particularly important to develop and promote specific guidelines and intervention measures aimed at improving the lifestyles of the older adults.

## Data Availability

The raw data supporting the conclusions of this article will be made available by the authors, without undue reservation.
